# Identification of Protective Antigens for Vaccination against Systemic Salmonellosis

**DOI:** 10.3389/fimmu.2014.00381

**Published:** 2014-08-11

**Authors:** Dirk Bumann

**Affiliations:** ^1^Focal Area Infection Biology, Biozentrum, University of Basel, Basel, Switzerland

**Keywords:** *Salmonella enterica*, protective immunity, mouse model, human clinical trials, antigen expression, immunodominance, typhoid vaccines

## Abstract

There is an urgent medical need for improved vaccines with broad serovar coverage and high efficacy against systemic salmonellosis. Subunit vaccines offer excellent safety profiles but require identification of protective antigens, which remains a challenging task. Here, I review crucial properties of *Salmonella* antigens that might help to narrow down the number of potential candidates from more than 4000 proteins encoded in *Salmonella* genomes, to a more manageable number of 50–200 most promising antigens. I also discuss complementary approaches for antigen identification and potential limitations of current pre-clinical vaccine testing.

## Introduction

*Salmonella enterica* serovars Typhi and Paratyphi A, B, and C cause human enteric fever with an estimated annual number deaths of 190,000 (1). Enteric fever disease burden is probably underestimated because of difficult and insensitive diagnosis methods (2). In addition to these serovars, specific strains of serovar Typhimurium, which usually causes self-limiting gastroenteritis, can also cause systemic disease, particularly in young HIV-infected children in sub-Saharan Africa (invasive non-typhoidal salmonellosis, iNTS) (3).

Enteric fever and iNTS become increasingly difficult to treat with antibiotics because of rising resistance to fluoroquinolones and cephalosporins, and new drug candidates for these and other Gram-negative pathogens are scarce suggesting a risk of an increasing number of untreatable cases (2, 4).

## The Need for Novel Subunit Vaccines

Enteric fever can be prevented with a variety of vaccines (5). Killed whole-cell preparations of serovars Typhi and Paratyphi were successfully used to diminish incidence in endemic areas, but their use was discontinued because of frequent adverse reactions (6). A live attenuated *S*. Typhi strain Ty21a that was generated by chemical mutagenesis confers a moderate level of protection for up to three years against serovar Typhi, but not other relevant serovars (6). Additional genetically modified *Salmonella* strains have been tested in clinical trials with some success, but none of them has yet reached approval. Finally, the purified capsular carbohydrate Vi of serovar Typhi induces protective immunity over several years against serovars Typhi (6) and possibly Paratyphi C, but not Paratyphi A and B or Typhimurium that all lack such a capsule. Conjugation of Vi with an unrelated protein antigen improves immune response in small infants, a major target population for enteric fever (6). To cover the important serovar Paratyphi A, current efforts focus on linking the O antigen (carbohydrate part of lipopolysaccharide) with a protein antigen (7).

In conclusion, treatment of systemic salmonellosis becomes increasingly difficult, and prevention with currently available vaccines is hampered by only moderate levels and limited duration of protection, and incomplete coverage of clinically relevant serovars. This situation generates an urgent medical need for improved *Salmonella* vaccines.

Live attenuated *Salmonella* strains offer important advantages such as low production costs and oral administration, but pose a risk of causing disease especially in immunocompromised patients that might be inadvertently exposed, e.g., household contacts of vaccines that shed live *Salmonella*. Whole-cell killed vaccines are effective but contain pyogenic components that cause unacceptable inflammatory responses. As a consequence, development focuses on subunit vaccines that contain one or several key antigens inducing protective immune responses.

The key challenge of developing such a vaccine is identification of suitable antigens. Unfortunately, among thousands of potential *Salmonella* antigen candidates, probably only very few have the necessary properties. Efficient strategies to identify protective antigens among large number of candidates have been developed and applied for vaccines that protect against extracellular pathogens using inhibitory/bactericidal antibodies (reverse vaccinology) (8). For these pathogens, suitable antigens need to be surface exposed to enable antibody binding, which substantially narrows down the number of potential candidates. Furthermore, immunization trials can be scored for inhibitory/bactericidal antibodies using rather simple assays amenable for high-throughput.

In contrast, similar strategies have not yet been developed for intracellular pathogens like *Salmonella* (which reside mostly in host macrophages during systemic disease), since criteria for pre-selecting promising antigens are unclear for most such pathogens, and immune correlates of protection remain poorly characterized. Antibodies (or just B cells) often contribute to protection but T cell responses are usually also required. The crucial αβ T cells recognize peptide epitopes and this led to a focus on protein antigens. Most intracellular pathogen genomes encode thousands of proteins, and identification of the few protective antigens among these numerous candidates remains challenging.

However, extensive recent work on *Salmonella* has uncovered some information that might be useful as a rational basis for future vaccine development against this and possibly other intracellular pathogens. In particular, coverage of relevant *Salmonella* strains, antigen expression in infected host tissues, and antigen compartmentalization within the *Salmonella* cell may substantially narrow down the number of promising antigen candidates.

## Antigens Enabling Broad Serovar Coverage

To achieve protective immunity against all relevant *Salmonella* strains, conserved antigens must be used. Hundreds of genes are missing or dysfunctional due to frameshift mutations or premature stop codons in certain relevant strains (9), but the rapidly increasing collection of genome sequences facilitates identification of suitable broadly conserved antigens. Orthologs usually share extensive sequence identity, but rare non-synonymous point mutations might still affect potentially crucial immunity determinants such as surface-exposed loops of outer membrane proteins (10). The 3D structures of many *Salmonella* proteins have been determined, and additional structures can be modeled based on homologs. However, it remains challenging to estimate which amino acid differences might impair cross-protective immune responses. As a consequence, antigens with highly conserved sequence among relevant serovars might be prioritized. On the other hand, antigens that play a potentially crucial role in pathogenesis of only a subset of serovars such as typhoid toxin (11) could still be an important contributor to vaccine combinations containing multiple antigens.

## Antigen Expression in Host Tissues

To detect and kill *Salmonella*, the immune system must recognize antigens that *Salmonella* expresses in infected host tissues. For animal infection models, purification of genetically engineered fluorescent *Salmonella* cells from infected tissue homogenates using flow cytometry yields sufficient material for large-scale proteome analysis (12, 13). The results reveal expression of more than 1800 *Salmonella* antigens in mouse spleen. As a caveat, this analysis misses most secreted *Salmonella* proteins that are lost during purification. This is important, since at least one secreted protein can confer moderate protection (14). While escaping proteomics of purified *Salmonella*, highly expressed secreted proteins can be identified based on transcriptional *in vivo* data (15, 16).

Recent advances in proteomics enable even absolute quantification of copy numbers per *Salmonella* cell for most detected antigens (10). High expression levels might facilitate immune recognition (10, 15), but our systematic analysis did not support that protective antigens are generally highly expressed (10). This could reflect extensive host–pathogen coevolution modulating expression levels and immunogenicity of antigens. However, despite the poor predictive power of quantitative expression levels, antigen expression itself remains a crucial precondition for protective immune responses.

*Salmonella* proteomes in human tissues have not yet been investigated. However, experimental infections of human volunteers have been done in the past (17, 18), and a well-controlled protocol has recently been established (19). Purification by flow cytometry similar to the mouse studies would require infection with a genetically modified *Salmonella* strain, and type and required quantities of biopsy material would need to be determined.

*Salmonella* virulence has been extensively characterized in the mouse typhoid fever model. These studies have identified more than 270 *Salmonella* genes that contribute to pathogenesis. In almost all cases, this evidence indicates expression of the respective antigens at least at some stage of the infection. Virulence phenotypes in human beings are also available in a few cases from vaccine trials with live attenuated *Salmonella* strains (20–22). These scarce human data are largely consistent with observations for the corresponding *Salmonella* mutants in the mouse model, but systematic comparisons are currently impossible due to the lack of human data for most potential virulence factors.

Using another indirect approach, large-scale studies have identified antibodies that specifically recognize dozens of *Salmonella* antigens in sera of acutely infected and convalescent patients or experimentally infected mice, but not uninfected controls (23, 24). The presence of such antibodies is a clear indication that the respective *Salmonella* antigens are expressed at least at some stages of infection. Interestingly, there is a considerable overlap in immune signature of murine and human salmonellosis. On the other hand, comparison with direct *ex vivo* proteome analysis of *Salmonella* purified from infected mouse spleen reveals that serum antibodies recognize only a small minority of the more than 1800 *in vivo* expressed *Salmonella* antigens. It is possible, that *Salmonella* antigens that induce specific antibodies are particularly accessible for the host immune system, and thus represent most promising vaccine antigen candidates. However, direct comparison of antibody titers in convalescent mice with antigen protectivity in immunization/challenge studies shows that serum antibody levels have poor predictive power for identifying suitable vaccine antigens (10, 23). In fact, several of the most protective vaccine antigens failed to elicit detectable antibody responses in both mice and human beings, while immunodominant antigens mostly fail to protect.

Similar to antibody response in convalescent individuals, T cell responses to specific *Salmonella* antigens provide information about antigen expression during infection. CD4 T cell epitopes have been comprehensively predicted based on peptide properties that facilitate binding to antigen-presenting major histocompatibility complex (MHC) II molecules and T cell receptors (25). Some antigens were experimentally confirmed to be recognized by T cells from infected human beings (26, 27) and mice (14, 25, 28, 29), but not uninfected individuals. These results confirmed expression of corresponding *Salmonella* antigens (including the promising antigen SseB) at least during some stages of infection. Again, these identified antigens are only a small subset of all expressed antigens, and T cell responses during infection have poor predictive power for protective antigens (10). This might reflect expression at infection stages (29) or in distinct tissue microenvironments (30) that have limited relevance for protective immunity.

In conclusion, proteomics and virulence phenotypes provide large-scale information on *Salmonella* antigen expression in infected mice. Together, some 2000 different *Salmonella* antigens are expressed during infection in the mouse typhoid fever model, and might thus represent potential vaccine antigens. Evidence for human infections is much more fragmentary and largely restricted to serum antibody and T cell responses.

## Antigen Compartimentalization

The localization of an antigen within the *Salmonella* cell may have a major impact on its protectivity. In particular, live intact *Salmonella* can only be detected by the host immune system through recognition of surface-exposed/released antigens, since internal *Salmonella* antigens are shielded by the cell envelope. On the other hand, dead *Salmonella* might release antigens regardless of their initial localization. In many infection foci, live and dead *Salmonella* reside in close proximity (30), and recognition of dead *Salmonella* alone might be sufficient for activation of bystander cells containing live *Salmonella*, resulting in effective clearance of both live and dead *Salmonella*. However, a subset of live *Salmonella* resides in tissue regions without any dead *Salmonella* (10, 30), and these would escape detection/clearance by immune responses directed exclusively against internal *Salmonella* antigens. This working model is supported by previously identified protective antigens (15, 31–33) and our systematic comparison of *Salmonella* antigens from different compartments (10): all identified protective antigens are surface exposed. A recent study extended this finding to a secreted virulence effector protein (14), supporting the hypothesis that antigens must be accessible on live *Salmonella* to confer protective immunity.

Surface exposure/secretion might represent a powerful criterion to narrow down the number of potentially promising *Salmonella* vaccine antigens. Surface-exposed outer membrane proteins can be identified based on primary sequence properties and have been tabulated in databases (34, 35). Interestingly, outer membrane-associated lipoproteins can also confer protective immunity, even when they likely localize to the shielded periplasmic side of the outer membrane (10). Possibly, such lipoproteins are released in outer membrane vesicles that are degraded in host cell lysosomes thus exposing lipoproteins to the antigen-presentation platforms. Outer membrane-associated lipoproteins can again be identified based on primary sequences (36). Experimental analysis of outer membrane preparations (37, 38) and/or biotinylated surface-exposed proteins (39) can be used to confirm theoretical predictions, and to identify additional exposed antigens that might be secreted through unconventional mechanisms.

In addition to surface-associated proteins, *Salmonella* translocates various proteins directly to the infected host cell cytosol, predominantly using the SPI-2 associated type III secretion system. SPI-2 effector proteins are intensively studied and the currently identified list of 32 proteins (40) might approximate completion. The SPI-2 translocon subunit SseB itself is one of the most promising vaccine antigens (15, 23, 27). During initial phases of infection, *Salmonella* secretes proteins also through the SPI-1 associated type III secretion system and through the flagellar apparatus (in particular, flagellin, a moderately protective antigen) (29, 31).

Together, surface-exposed and secreted *Salmonella* antigens comprise some 200 different antigens, and at least around 50 of them are expressed during infection in the mouse typhoid model based on transcriptional data, proteomics, virulence phenotypes, and/or immunization data. Twenty-six such antigens have already been tested and nine appear to confer some degree of protective immunity in mouse typhoid fever immunization/challenge studies (FliC, SseB, OmpD, CirA, IroN, T0937, SlyB, PagN, and SseI; in some cases group sizes were too small to obtain definitive proof) (10, 14, 15, 29, 31–33).

## Future Perspectives

Immunization/challenge experiments in the mouse typhoid fever model have shown that live attenuated *Salmonella* strains can provide full long-term protection against otherwise lethal challenge infections (41). Compared to this benchmark, progress with subunit vaccines in the same model has been somewhat disappointing. Despite large-scale experimental and computational screening campaigns in several different laboratories, few *Salmonella* antigens with at most moderate protectivity in the mouse typhoid fever model have been identified. None of these antigens confer full protection for more than some 30 days after challenge infection. This could reflect immune evasion of the challenge *Salmonella* strain by mutation of crucial epitopes within the respective antigens. During such a long infection time, other adaptive immune responses might be expected, but these responses are obviously insufficient for protective immunity.

It is possible that the best protective antigens have not yet been identified, or that multiple antigens need to be combined for full protection and prevention of immune evasion. It is also possible that antigens other than proteins, such as lipids, carbohydrates, or even small molecules (42), are necessary for high levels of protection. One approach to test this hypothesis could use progressive depletion of specific antigens from protective live attenuated *Salmonella* strains, by deleting respective biosynthesis genes. However, this approach is limited to non-essential genes and is thus non-informative for antigens such as riboflavin intermediates (12, 42). Alternatively, killed whole-cell vaccines might be fractionated and tested for protection. Unfortunately, killed whole-cell vaccine formulations with high protective efficacy in the mouse typhoid fever model have not yet been described. Future studies might revisit this issue, especially since killed whole-cell vaccines confer substantial protective immunity against invasive salmonellosis in human beings (although they are no longer used because of severe adverse reactions) (1).

Finally, it is important to consider what level of protection is actually needed in pre-clinical mouse models before proceeding to human clinical vaccine trials. In the typhoid fever model, genetically high-susceptible mouse strains defective for the divalent cation transporter *Slc11a1* (*NRAMP1*) (43), are infected with doses of *S. enterica* serovar Typhimurium that result in an attack rate of 100%. This combination reproduces some important aspects of human disease including *Salmonella* dissemination from intestinal sites, histopathology in spleen and liver (splenomegaly, formation of structured inflammatory lesions), relevance of various *Salmonella* virulence factors and host cytokines, and protective immunity against reinfection in convalescent individuals, or individuals vaccinated with live attenuated *Salmonella* strains (41). On the other hand, disease progression in mice is more rapid and always lethal when using wild-type *Salmonella* strains, in contrast to human enteric fever. Importantly, protective immunity against challenge infections in the mouse model requires both B cell and CD4 T cell responses (28), but neither antibodies (44) nor MHC I-restricted CD8 T cells (45). In contrast, vaccination-induced antibodies alone seem to confer already a substantial level of protection in human beings (46), at least in endemic areas where pre-existing immune responses to *Salmonella* are highly prevalent (24). It is thus possible that full protection against virulent wild-type *Salmonella* strains in genetically susceptible mice might be too stringent a criterion to judge vaccine efficacy.

Instead, it might be worth considering genetically resistant mice (47), in which antibodies seem to suffice for protective immunity (48), and heat-killed *Salmonella* mediate substantial immunity (49) similar to the situation in human beings (6). Interestingly, flagellin is also highly protective in resistant mice (50), in contrast to only moderate protectivity in susceptible mice.

In addition to the mouse strain, the challenge infection dose should be re-considered. Controlled human trials have shown that vaccine-induced immunity can be easily overwhelmed by even moderate challenge doses (17). Vaccines that have well-documented efficacy in field-trials, completely fail to protect against *S*. Typhi when given at doses in the range of 10^6^–10^7^ CFU. Vaccine efficacy is only seen at a much lower dose of 10^5^ CFU that caused disease in only 40% of unvaccinated control volunteers [a recent study showed higher attack rates at such doses (19)]. Based on these human data, commonly used mouse challenge infections that result in 100% attack rates might be too stringent for revealing a moderate level of protective immunity that could still be sufficient for preventing even a large proportion of human disease under relevant field conditions.

Finally, a better understanding of human immune responses that are relevant for protective immunity could help to replace the crude readout parameter “survival after challenge infection” with more informative quantitative immune parameters. Ongoing studies in an experimental human infection and vaccination model (19) will likely provide such crucial information in the near future.

## Conclusion

Several *Salmonella* antigens that can mediate at least partial protective immunity against lethal challenge infections in mice have recently been identified. Analysis of their properties suggests that efforts to identify further suitable antigens might focus on a limited number of promising surface-associated/secreted candidates that are expressed in infected host tissues. However, none of the known individual antigens mediates solid strong protection, comparable to what can be achieved with attenuated live *Salmonella* strains. Future studies could explore antigen combinations and possibly antigens other than proteins. Moreover, a better understanding of qualitative and quantitative immune parameters that are required to protect human beings is needed to guide pre-clinical models for further vaccine optimization and to determine what levels of protection are needed.

## Conflict of Interest Statement

The author declares that the research was conducted in the absence of any commercial or financial relationships that could be construed as a potential conflict of interest.

## References

[1] Lozano R Naghavi M Foreman K Lim S Shibuya K Aboyans V Global and regional mortality from 235 causes of death for 20 age groups in 1990 and 2010: a systematic analysis for the Global Burden of Disease Study 2010. Lancet (2012) 380:2095–128 10.1016/S0140-6736(12)61728-0 23245604 PMC10790329

[2] Crump JA Mintz ED Global trends in typhoid and paratyphoid fever. Clin Infect Dis (2010) 50:241–6 10.1086/649541 20014951 PMC2798017

[3] Maclennan CA Levine MM Invasive nontyphoidal *Salmonella* disease in Africa: current status. Expert Rev Anti Infect Ther (2013) 11:443–6 10.1586/eri.13.27 23627848

[4] Jean SS Hsueh PR High burden of antimicrobial resistance in Asia. Int J Antimicrob Agents (2011) 37:291–5 10.1016/j.ijantimicag.2011.01.009 21382699

[5] Martin LB Vaccines for typhoid fever and other salmonelloses. Curr Opin Infect Dis (2012) 25:489–99 10.1097/QCO.0b013e328356ffeb 22825288

[6] Anwar E Goldberg E Fraser A Acosta CJ Paul M Leibovici L Vaccines for preventing typhoid fever. Cochrane Database Syst Rev (2014) 1:CD001261 10.1002/14651858.CD001261.pub3 24385413

[7] Maclennan CA Martin LB Micoli F Vaccines against invasive *Salmonella* disease: current status and future directions. Hum Vaccin Immunother (2014) 10:33–48 10.4161/hv.29054 24804797 PMC4185946

[8] Sette A Rappuoli R Reverse vaccinology: developing vaccines in the era of genomics. Immunity (2010) 33:530–41 10.1016/j.immuni.2010.09.017 21029963 PMC3320742

[9] Nuccio SP Baumler AJ Comparative analysis of *Salmonella* genomes identifies a metabolic network for escalating growth in the inflamed gut. MBio (2014) 5:e929–914 10.1128/mBio.00929-14 24643865 PMC3967523

[10] Barat S Willer Y Rizos K Claudi B Maze A Schemmer AK Immunity to intracellular salmonella depends on surface-associated antigens. PLoS Pathog (2012) 8:e1002966 10.1371/journal.ppat.1002966 23093937 PMC3475680

[11] Song J Gao X Galan JE Structure and function of the *Salmonella* Typhi chimaeric A(2)B(5) typhoid toxin. Nature (2013) 499:350–4 10.1038/nature12377 23842500 PMC4144355

[12] Becker D Selbach M Rollenhagen C Ballmaier M Meyer TF Mann M Robust *Salmonella* metabolism limits possibilities for new antimicrobials. Nature (2006) 440:303–7 10.1038/nature04616 16541065

[13] Steeb B Claudi B Burton NA Tienz P Schmidt A Farhan H Parallel exploitation of diverse host nutrients enhances *Salmonella* virulence. PLoS Pathog (2013) 9:e1003301 10.1371/journal.ppat.1003301 23633950 PMC3636032

[14] Kurtz JR Petersen HE Frederick DR Morici LA Mclachlan JB Vaccination with a single CD4 T cell peptide epitope from a *Salmonella* type III-secreted effector protein provides protection against lethal infection. Infect Immun (2014) 82:2424–33 10.1128/IAI.00052-14 24686055 PMC4019158

[15] Rollenhagen C Sorensen M Rizos K Hurvitz R Bumann D Antigen selection based on expression levels during infection facilitates vaccine development for an intracellular pathogen. Proc Natl Acad Sci U S A (2004) 101:8739–44 10.1073/pnas.0401283101 15173591 PMC423265

[16] Rollenhagen C Bumann D *Salmonella enterica* highly expressed genes are disease specific. Infect Immun (2006) 74:1649–60 10.1128/IAI.74.3.1649-1660.2006 16495536 PMC1418657

[17] Woodward WE Volunteer studies of typhoid fever and vaccines. Trans R Soc Trop Med Hyg (1980) 74:553–6 10.1016/0035-9203(80)90133-9 7210105

[18] Levine MM Tacket CO Sztein MB Host–*Salmonella* interaction: human trials. Microbes Infect (2001) 3:1271–9 10.1016/S1286-4579(01)01487-3 11755415

[19] Waddington CS Darton TC Jones C Haworth K Peters A John T An outpatient, ambulant-design, controlled human infection model using escalating doses of *Salmonella* Typhi challenge delivered in sodium bicarbonate solution. Clin Infect Dis (2014) 58:1230–40 10.1093/cid/ciu078 24519873 PMC3982839

[20] Bumann D Hueck C Aebischer T Meyer TF Recombinant live *Salmonella* spp. for human vaccination against heterologous pathogens. FEMS Immunol Med Microbiol (2000) 27:357–64 10.1111/j.1574-695X.2000.tb01450.x 10727892

[21] Tacket CO Levine MM CVD 908, CVD 908-htrA, and CVD 909 live oral typhoid vaccines: a logical progression. Clin Infect Dis (2007) 45(Suppl 1):S20–3 10.1086/518135 17582563

[22] Frey SE Lottenbach KR Hill H Blevins TP Yu Y Zhang Y A Phase I, dose-escalation trial in adults of three recombinant attenuated *Salmonella* Typhi vaccine vectors producing *Streptococcus pneumoniae* surface protein antigen PspA. Vaccine (2013) 31:4874–80 10.1016/j.vaccine.2013.07.049 23916987

[23] Lee SJ Liang L Juarez S Nanton MR Gondwe EN Msefula CL Identification of a common immune signature in murine and human systemic Salmonellosis. Proc Natl Acad Sci U S A (2012) 109:4998–5003 10.1073/pnas.1111413109 22331879 PMC3324033

[24] Liang L Juarez S Nga TV Dunstan S Nakajima-Sasaki R Davies DH Immune profiling with a *Salmonella* Typhi antigen microarray identifies new diagnostic biomarkers of human typhoid. Sci Rep (2013) 3:1043 10.1038/srep01043 23304434 PMC3540400

[25] Maybeno M Redeker A Welten SP Peters B Loughhead SM Schoenberger SP Polyfunctional CD4(+) T cell responses to immunodominant epitopes correlate with disease activity of virulent *Salmonella*. PLoS One (2012) 7:e43481 10.1371/journal.pone.0043481 22912884 PMC3422266

[26] Sheikh A Khanam F Sayeed MA Rahman T Pacek M Hu Y Interferon-gamma and proliferation responses to *Salmonella enterica* serotype Typhi proteins in patients with *S*. Typhi Bacteremia in Dhaka, Bangladesh. PLoS Negl Trop Dis (2011) 5:e1193 10.1371/journal.pntd.0001193 21666798 PMC3110156

[27] Reynolds CJ Jones C Blohmke CJ Darton TC Goudet A Sergeant R The serodominant secreted effector protein of *Salmonella*, SseB, is a strong CD4 antigen containing an immunodominant epitope presented by diverse HLA class II alleles. Immunology (2014):10.1111/imm.12327 24891088 PMC4212957

[28] Cookson BT Bevan MJ Identification of a natural T cell epitope presented by *Salmonella*-infected macrophages and recognized by T cells from orally immunized mice. J Immunol (1997) 158:4310–9 9126993

[29] Lee SJ Mclachlan JB Kurtz JR Fan D Winter SE Baumler AJ Temporal expression of bacterial proteins instructs host CD4 T cell expansion and th17 development. PLoS Pathog (2012) 8:e1002499 10.1371/journal.ppat.1002499 22275869 PMC3262010

[30] Burton NA Schurmann N Casse O Steeb AK Claudi B Zankl J Disparate impact of oxidative host defenses determines the fate of *Salmonella* during systemic infection in mice. Cell Host Microbe (2014) 15:72–83 10.1016/j.chom.2013.12.006 24439899

[31] McSorley SJ Cookson BT Jenkins MK Characterization of CD4+ T cell responses during natural infection with *Salmonella* Typhimurium. J Immunol (2000) 164:986–93 10.4049/jimmunol.164.2.986 10623848

[32] Gil-Cruz C Bobat S Marshall JL Kingsley RA Ross EA Henderson IR The porin OmpD from nontyphoidal *Salmonella* is a key target for a protective B1b cell antibody response. Proc Natl Acad Sci U S A (2009) 106:9803–8 10.1073/pnas.0812431106 19487686 PMC2701014

[33] Yang Y Wan C Xu H Aguilar ZP Tan Q Xu F Identification of an outer membrane protein of *Salmonella enterica* serovar Typhimurium as a potential vaccine candidate for Salmonellosis in mice. Microbes Infect (2013) 15:388–98 10.1016/j.micinf.2013.02.005 23485513

[34] Paramasivam N Linke D ClubSub-P: cluster-based subcellular localization prediction for Gram-negative bacteria and archaea. Front Microbiol (2011) 2:218 10.3389/fmicb.2011.00218 22073040 PMC3210502

[35] Freeman TC Jr. Wimley WC TMBB-DB: a transmembrane beta-barrel proteome database. Bioinformatics (2012) 28:2425–30 10.1093/bioinformatics/bts478 22843985 PMC3463127

[36] Diaz-Mejia JJ Babu M Emili A Computational and experimental approaches to chart the *Escherichia coli* cell-envelope-associated proteome and interactome. FEMS Microbiol Rev (2009) 33:66–97 10.1111/j.1574-6976.2008.00141.x 19054114 PMC2704936

[37] Chooneea D Karlsson R Encheva V Arnold C Appleton H Shah H Elucidation of the outer membrane proteome of *Salmonella enterica* serovar Typhimurium utilising a lipid-based protein immobilization technique. BMC Microbiol (2010) 10:44 10.1186/1471-2180-10-44 20149234 PMC2829538

[38] Brown RN Sanford JA Park JH Deatherage BL Champion BL Smith RD A comprehensive subcellular proteomic survey of *Salmonella* grown under phagosome-mimicking versus standard laboratory conditions. Int J Proteomics (2012) 2012:123076 10.1155/2012/123076 22900174 PMC3410353

[39] Sabarth N Lamer S Zimny-Arndt U Jungblut PR Meyer TF Bumann D Identification of surface-exposed proteins of *Helicobacter pylori* by selective biotinylation, affinity purification, and two-dimensional gel electrophoresis. J Biol Chem (2002) 277:27896–902 10.1074/jbc.M204473200 12023975

[40] Figueira R Watson KG Holden DW Helaine S Identification of *Salmonella* pathogenicity island-2 type III secretion system effectors involved in intramacrophage replication of *S. enterica* serovar typhimurium: implications for rational vaccine design. MBio (2013) 4:e00065 10.1128/mBio.00065-13 23592259 PMC3634603

[41] Dougan G John V Palmer S Mastroeni P Immunity to salmonellosis. Immunol Rev (2011) 240:196–210 10.1111/j.1600-065X.2010.00999.x 21349095

[42] Corbett AJ Eckle SB Birkinshaw RW Liu L Patel O Mahony J T-cell activation by transitory neo-antigens derived from distinct microbial pathways. Nature (2014) 509:361–5 10.1038/nature13160 24695216

[43] Valdez Y Ferreira RB Finlay BB Molecular mechanisms of *Salmonella* virulence and host resistance. Curr Top Microbiol Immunol (2009) 337:93–127 10.1007/978-3-642-01846-6_4 19812981

[44] Nanton MR Way SS Shlomchik MJ McSorley SJ Cutting edge: B cells are essential for protective immunity against *Salmonella* independent of antibody secretion. J Immunol (2012) 189:5503–7 10.4049/jimmunol.1201413 23150714 PMC3518619

[45] Lee SJ Dunmire S McSorley SJ MHC class-I-restricted CD8 T cells play a protective role during primary *Salmonella* infection. Immunol Lett (2012) 148:138–43 10.1016/j.imlet.2012.10.009 23089550 PMC3540194

[46] Strugnell RA Scott TA Wang N Yang C Peres N Bedoui S *Salmonella* vaccines: lessons from the mouse model or bad teaching? Curr Opin Microbiol (2014) 17:99–105 10.1016/j.mib.2013.12.004 24440968

[47] Simon R Tennant SM Galen JE Levine MM Mouse models to assess the efficacy of non-typhoidal *Salmonella* vaccines: revisiting the role of host innate susceptibility and routes of challenge. Vaccine (2011) 29:5094–106 10.1016/j.vaccine.2011.05.022 21616112 PMC3152302

[48] Eisenstein TK Angerman CR Immunity to experimental *Salmonella* infection: studies on the protective capacity and immunogenicity of lipopolysaccharide, acetone-killed cells, and ribosome-rich extracts of *Salmonella typhimurium* in C3H/HeJ and CD-1 mice. J Immunol (1978) 121:1010–4 690428

[49] Herzberg M Nash P Hino S Degree of immunity induced by killed vaccines to experimental salmonellosis in mice. Infect Immun (1972) 5:83–90 4570987 10.1128/iai.5.1.83-90.1972 PMC422325

[50] Simon R Tennant SM Wang JY Schmidlein PJ Lees A Ernst RK *Salmonella enterica* serovar enteritidis core O polysaccharide conjugated to H:g,m flagellin as a candidate vaccine for protection against invasive infection with S. enteritidis. Infect Immun (2011) 79:4240–9 10.1128/IAI.05484-11 21807909 PMC3187246

